# Building Your Dream Team: How Indie Teams Can Form, and Thrive Together

**DOI:** 10.12688/f1000research.139274.1

**Published:** 2024-01-08

**Authors:** Minhaz-Us-Salakeen Fahme, Afia Adiba

**Affiliations:** 1 Battery Low Interactive Ltd., Dhaka, 1208, Bangladesh

**Keywords:** Indie team, head-hunting, people management, communication, team chemistry, conflict resolution

## Abstract

The majority of indie game development teams are driven by the desire to produce a blockbuster game. But there are many obstacles on the way to realizing this desire, especially in the beginning. It is possible to create a game alone, but more often than not, something genuinely amazing involves a group of talented people working together toward a similar objective.

Finding the correct stakeholders is one of the biggest problems with starting an independent game development team. People with the required technical talents, such as programmers and artists, as well as those with business acumen, like marketing and finance experts, are included in this. Success depends on being able to locate these people and manage them. But after the correct individuals have been identified, it is crucial to develop open lines of communication and a common comprehension of the project’s objectives and expectations.

Finding the perfect people to join their team is a problem that independent game development teams frequently encounter. Attracting talented individuals to a brand-new, unproven initiative might be difficult. The attention of skilled people interested in working on cutting-edge and intriguing initiatives might be attracted through networking.

There are other things to consider as well since forming a team comes with the type of game one is making, finding skilled teammates, team chemistry, conflict resolution, communication, long-term relation maintenance and leadership aspects.

In general, creating and leading an independent game team is a difficult process. There is no specific method for doing this, but with a little help from others’ expertise, it is possible to assemble a team of creative people who can collaborate to produce a game that is genuinely enjoyable.

## 1. Introduction

The gaming industry has undergone a considerable transition in recent years toward indie game creation. Globally, the number of independent game studios has grown significantly in numerous nations as a result of the democratization of the game production industry ( fungies.io, 2023). While the majority of independent game creators are located in the United States, United Kingdom, Canada, and Australia, there are a number of developing nations that are making their mark in the sector. These nations include Kazakhstan, Poland, India, and Romania ( fungies.io, 2023). This development has given game creators the ability to produce profitable games without the need for financial assistance from publishers, opening up new possibilities for aspirant game developers to realize their vision for a game with a group of independent developers. Since game creation requires numerous jobs that are often not carried out by a single person, collaboration is essential for success. To maximize the likelihood of success, it is imperative to find others who possess the relevant skills. In light of this, it is important to take into account the common positions held by programmers, artists, and designers in independent game production teams. Even if a person is not interested in starting their own team, knowing the important roles in independent teams can help them concentrate on honing their talents and better prepare for possibilities to join another team. The global market for independent video games is predicted to grow at an unprecedented rate, reaching $305 billion by 2025 ( Global Data). A growing number of developers who want to leave their imprint on the business are being drawn in by this expansion. Many independent (indie) game developers understand the need for a skilled team to realize their vision, even though others may want to work alone, but creating and leading a team may be a difficult task.

The advantages of developing an indie game have been a topic of interest in the gaming industry for the past few years. Contrary to major corporations, independent game developers typically work in teams of up to 10 to create indie games. Despite not always referring to games created by amateurs, the phrase “indie game” is typically used to describe games that are purposefully produced and disseminated using techniques different from those employed by the large video game companies ( Lipkin, 2012). To exemplify for better context, first, a strike team is a small, independent squad. Strike teams, such as Special Weapons and Tactics (SWAT) teams, rescue teams, and firefighters, are groups that perform tasks in perilous and time-critical circumstances in real world. In traditional game development, the term ‘strike team’ is frequently used to describe an unplanned small team formed within a bigger team to finish a specific difficult task. According to a Quora post by a game developer who has worked on both indie and AAA (not formal classification, refers to high profile high budget projects) projects, “these teams are small, and thanks to their interdisciplinary structure and a fluid information flow they can quickly deal with an issue” ( Freeman *et al*., 2020). These games rely primarily on word-of-mouth advertising, social media, streaming platforms, and YouTube to promote their games because they lack the financial backing and extensive marketing teams of major game developers ( The rise of indie games). Independent game creators, however, can benefit from greater creative flexibility, flexible work schedules, and an opportunity to advance their careers with a smaller team. Surveys were done on why people would prefer to join an independent team by Freeman and McNeese (2019), and some noteworthy responses will be quoted here: one participant stated “I’ve found the indie field to be filled with extremely friendly, creative, and passionate people, more so than any other industry. It is very small and tight knit. Indies tend not to see each other as competitors, and always do whatever they can to help each other get that big break.” Another participant pointed out a very significant reason to join an indie team; “Independence really boils down to a game creator that owns their creation and intellectual property. They aren’t making someone else’s vision or owned by a publisher. Indies can work with publishers and the typical investment thing happens where a publisher will take some of the profits, but in large part, the indie owns what they make.” Independent game development is a fantastic approach for recent graduates or those new to programming to broaden their skills. How can one make their ambitions of creating independent video games a reality?

The number of independent game developers has increased dramatically in recent years as they seek to produce original games. According to recent polls, the majority of independent game developers prefer to work in teams. It is found in an International Game Developers Association (IGDA) poll that 62% of independent game developers prefer to work in teams of between two and 10 individuals, while 47% prefer to work in teams of two to five ( The International Game Developers Association (IGDA) Developer Satisfaction Survey 2019 Summary Report, 2023). According to the same poll, programming, art, and design are the most prevalent roles in these teams. Some of the most frequently claimed benefits according to this study of working in a team are collaboration, merging complementing skill sets, and burden sharing. IGDA Developer Satisfaction Survey 2019 found that while the industry still lacks gender diversity, indie game development teams are typically more diverse in terms of age and experience, with 33% of indie game developers being aged 25-34 and 41% having less than five years of industry experience. The average cost of creating a mobile game is $3000 to $150000 depending on the sort of game ( HitBerry Games), but managing an independent game production team can also be pricey. According to a survey by the Game Developers Conference (GDC), 63% of independent game creators rely on their own personal funds as their main source of income. With these difficulties and possibilities in mind, it is critical for independent game developers to comprehend how to assemble and lead a productive team that can realize their vision. Nevertheless, team communication and management remain the biggest challenges for 53% of developers, according to the IGDA survey.

Establishing and managing the teams provide particular difficulties for independent game development teams. At the Game Developers Conference 2015, there took place a Producer Panel: Managing Your Indie Team and the speakers were Kara Kono, Jenna Hoffstein, Aaron Isaksen, Alex Schwartz and Amy Dallas. who examined the difficulties independent teams encounter while forming productive game development teams (GDC, 2015, Link: https://www.gdcvault.com/play/1022225/Producer-Panel-Managing-Your-Indie). They emphasize how crucial team dynamics, cooperation, and communication are to succeed in the independent game production sector. In order to promote creativity and productivity, the study underlines the need for a diversified skillset within the team as well as effective management tactics.

From my experience in the industry, it is exciting to build an independent team. The process of developing an independent game can be made or broken by choosing the correct team. Sifting through potential team members can be difficult due to the enormous talent pool available. From the several involvements in the indie team, it was observed that as soon as the enthusiasm sets in for being a part of an indie team, a ton of questions, uncertainties, and decision-making points appear like a cloud in the mind. This article delves into the complex world of managing and growing indie teams, covering the issues, tactics, and tensions that arise. For aspiring independent team owners and managers, insights and helpful suggestions are offered, drawing on actual experiences and published literature. Emphasizing the significance of effective communication and conflict resolution in promoting a healthy team culture, the main competencies and traits required for creating effective teams are reviewed. Additionally, the particular difficulties experienced by independent teams are discussed along with suggested solutions. By the end of this piece, it is our aim that readers will have a better understanding of the dynamics inside independent teams and have gained useful information for navigating the fascinating but challenging world of independent game development.

The whole process can be divided into three phases: laboratory (solo work), hunt (talent finding), and orchestra (maintenance of the team). The names of the phases were taken from personal experiences, and it is important to note that an effort was made to cover the bulk of points within these three stages in order to improve comprehension. The procedures described in this article have been tested in real-world settings where they were functioning as independent teams. Furthermore, it is important to note that some steps correspond to those that have been reported by various authors in previous literature.

## 2. Laboratory

The precise project requirements, including team composition, required tasks, and areas where assistance is needed, are identified at this point. It is easy to get paralyzed by doubt, which breeds indecision. However, one can get the answers to these queries such as the skill sets that are crucial, what type of games are suitable for the team to work etc. in a methodical way in the following section. Making judgments concerning the independent team can be done in a more informed way by using this approach. The initial stage entails determining the project’s core. The following queries should be taken into account:

### 2.1 What skill sets should one have?

To determine the necessary team members for indie game development, the first step is identifying the core skills required for the task. There are some primary questions that should be asked to determine personal skill sets. The questions include determining one’s strengths in game design, coding, or concept art. One should also rate themselves honestly and list the skill sets that are almost acquired but untested. This will assist in identifying the areas where support is needed, and the types of team members required.

### 2.2 What does one want to make?

It is crucial to decide what kinds of games the group intends to generate after establishing an independent game production studio. Being realistic about what can be accomplished right now is equally vital as having a good understanding of the genre and style of the game. Making something big may not be possible right away, therefore the team should have a long-term plan on how to get there. Setting up checkpoints along the way will not only keep the crew on task but also offer them a feeling of purpose and direction. Is it a roguelike metroidvania or turn-based card game, or something else? To get good support, Travis Tracy wrote in his article “ How to Build a Game Development Team” that it is essential to have a clear idea of the game. People will be unwilling to join the initiative without a clear plan in place.

Tracy also mentioned that talented people and good developers look for initiatives that catch their attention. They have the skills, and there are many chances for them because many people are engaged in highly imaginative initiatives and many of them require help. If one does not already have experience in game development or anything to offer, they will not be able to attract them. In this situation, a prepared Game Design Document (GDD: a highly descriptive software design document for a video game design) can assist in assisting others in understanding what someone is attempting to produce. Therefore, it is unquestionably preferable to be ready with a GDD that can be shared.

### 2.3 Why does one want to make it?

In game creation, it is essential to comprehend the project’s motives and objectives. The game’s creator needs to consider their goals and what they hope to accomplish with it. It is crucial to pose pointed queries like: why did you select this specific genre or game? Is there a message or subject that must be covered? Why is this significant and what the developer hopes to achieve specifically? Early responses to these queries could aid the developer in staying focused on their objective.

### 2.4 Ideally, what skillsets are needed to make it happen?

Depending on the abilities needed for the game, a basic estimation should be made. It is important to take into account aspects like the genre-specific art requirements, the complexity of the programming, and whether a lengthy plot or intricate gameplay is necessary. The precise skills needed for the game to succeed must be determined and listed. Prioritize the fundamental abilities to ensure that the primary knowledge is current to prevent any potential skill gaps. According to Sennett (2008), a skill is nothing more than a taught practice. It can include the knowledge, skills, and abilities employed in producing something as well as the formal knowledge and skills needed for the work ( Cockburn, 1983). A skill can only be developed *via* practice that is ingrained in routine. A person can train, develop, and eventually master a set of talents through organized repetition, from which they can start expanding even further.

If someone is thinking about forming their own independent game development team, an indie team might typically have certain common duties. Though some of them might be disregarded depending on the game, such as if it lacks text or music, nearly all of them are abilities that should be taken into account to raise the possibility that your game will be a success ( New York Film Academy, 2014). The essential team member is programmers - without a developer, even the best concept someone could come up with would probably never be put into action. The individual who will spend the majority of their time developing the game’s tools and codebase is a programmer. It should be mentioned that a programmer often performs more than only developing the game’s code in an independent team. They will also be responsible for beta testing and problem-fixing to make sure the game is polished and playable. Then there is the role of the game designer, who is in charge of level design, stories, and gameplay mechanics. Characters, backdrops, and user interfaces are examples of the visual assets that artists are in charge of generating. Musician/Sound Designer: this person is in charge of coming up with the game’s music and sound effects. The storyline and dialogue for the game are created by writers. Bugs must be found and reported by testers in order for the game to be fun and user-friendly. Each of these responsibilities is essential to the growth process and calls for a particular set of abilities. In order to fill each of these jobs effectively, an indie team should ideally consist of persons with a variety of skill sets ( gamedeveloper.com, 2010). It is crucial to remember that not all independent teams will have people in each of these positions. Members of smaller teams could take on several roles and responsibilities. This might cause problems like uneven workload distribution and exhaustion, but it can also encourage teamwork and adaptability.

Cross-referencing the requirements with the available inventory can then assist in evaluating whether the required resources are sufficient to complete the operation. The essential elements required to bring the dream to life have already been discovered. Is the current inventory, however, sufficient to me *et al*l the requirements, or will further support be required? Even if all the required skillsets are there to develop the game, or at least the initial iteration, this does not imply that the aim has been met. Additional services like quality assurance, publisher pitching, and business assistance are also required. Making a game for leisure may be the greatest solution if no one is managing the business element of game development. It is critical to have someone in charge of making sure the game is lucrative. The entire team must remember that they are designing a game for business goals, not only for entertainment. This encompasses marketing, sales, developing business strategies, and maybe securing money. If the individual is unable to complete these activities on their own, they can hire or work with outside expertise. Despite the fact that many business-to-business (B2B) organizations provide these services, they must be managed. If one can make it this far without incident, it can be presumed that they are confident enough to go on their own. The final barrier, however, is the individual. Game creation can be daunting, especially if you want to be successful. Having a spouse can make a major impact during difficult times. However, if the individual is confident enough to tackle these problems alone, they may go alone. There are several tools available to aid them in the age of artificial intelligence (AI).

## 3. Hunt

If any of the previous milestones were not completed, it is time to seek outside assistance, in the phase we call the hunt. The next stage is to look for qualified team members. There are numerous resources available at conferences and forums to help you identify qualified applicants. However, how does one go about finding the ideal person for their team? Furthermore, how can they be persuaded to join the project? These difficulties can be overcome with good planning which are briefly discussed in the next section.

### 3.1 Narrate your origin story

One of the most important jobs completed in the laboratory section (please see Section 2 Laboratoy) was answering vital questions such as the mission and the driving force behind it. It is critical that this information be shared with others. In the video game industry, execution is more crucial than having a great idea. Even a good idea cannot provide good outcomes unless it is properly executed. As a result, there is a need to overcome anxiety and confidently conveying the creation story of the game idea to the team members. The best method to attract others who share your values is to express your original thoughts. It is not necessary to rely entirely on monetary incentives or celebrities to inspire people to join a project; staying focused on a single objective can be just as effective. If one’s luck is on their side, they may come across others who share their level of passion, energy, and commitment to their cause. It is critical to publicize one’s mission and make it known to both physical and virtual groups. Speaking about it at social occasions may result in people recommending appropriate candidates. Although job postings may generate a huge number of applicants, it is still beneficial to use personal contacts to uncover talent. Personal relationships can be an excellent resource for locating persons with the necessary skills and expertise. It is possible to uncover potential team members that are suited for the project by employing one’s network, as well as to use pre-existing relationships to generate trust and cultivate relationships from the start. This strategy can be especially useful in the early phases of team creation when you are attempting to lay a firm basis for the team’s success. Sharing your story with potential teammates can also provide you with additional benefits. It may help in attracting people with similar perspectives. People are drawn to people, so this is your chance to put yourself out there and be regarded as someone with whom they can share their thoughts and ideas. This tiny distinction may be all that is required in critical situations.

After selecting potential team members, it is critical to be open and honest about the project’s goals, schedule, and remuneration plan. You should also outline the advantages of joining the team. Additionally, the time each team member can devote to the project should be discussed and decided upon beforehand. To create a cohesive team, it is critical to demand the same amount of dedication from each member. Finding the appropriate talent is important, but building a team also requires assembling a group of people who get along well and are dedicated to the game’s success.

Good game development requires a number of technical skills, including modeling and programming. The most crucial need for maintaining their technological methods, according to many independent developers, is “shared passion,” not technical proficiency ( Freeman *et al*., 2020). For example, an online post on the indie gamer forum explained how a shared passion was central to indie game development: “You need to find people who are passionate about creating a vision together and want to bring their strengths in to make it happen. Over the course of time, you then have to accept that this team will have highs and lows, and people come and go, but with a strong enough core team who shares the same passion, you should still be fine.”

### 3.2 David or Goliath?

One may struggle with the decision of whether to hire and train recent graduates or seasoned experts when it comes to hiring for the team. Each choice offers benefits and drawbacks of its own. Although employing an enthusiastic and motivated rookie might take more time at first, it might be worthwhile in the long term. Freshmen are more adaptable to alter and simpler to shape to the needs of the project. They are a significant asset since they are driven to achieve and eager to show themselves. Furthermore, showing them respect can result in enduring loyalty. However, on the other hand, seasoned experts could demand greater pay and not be as adaptable to change but can contribute a wealth of knowledge and skills to the team. Building a solid basis for a long-term team requires a mix of inexperienced and experienced team members. Hiring inexperienced employees necessitates a time commitment in training and molding them, which can occasionally result in budget and time overrun. Inexperienced persons frequently lack perception at first. Experienced campaigners, on the other hand, require little or no training and can give a variety of perspectives according to their backgrounds. However, because their ideologies and mentalities are already set, experienced campaigners can be difficult to influence instantly. This isn’t necessarily a bad thing, as their differences can help the cause significantly. They can, however, be tough to work with at times. Furthermore, recruiting a veteran increases the risk of bringing on someone with negative energy compared to hiring a novice. As a result, while recruiting an experienced applicant, it is critical to look for warning signs such as massive cultural differences, not being able to change mindset, rigid attitudes, predetermined inferiority/superiority complex. We have observed that cultural differences can be worked on with efforts however it gets hard to work with someone who is not ready to work with an open mind. However, it is not suggested to hire exclusively novices because they will require experienced team members to guide them.

For an independent game studio, choosing whether to use internal or external resources might be difficult. A team can access a variety of specialized talents and knowledge through outsourcing, which can also lower expenses and lighten the strain on in-house team members. However, it can also lead to management and communication problems because of time zone, linguistic, and cultural disparities.

Using internal resources, on the other hand, offers the benefit of working with a team that is already familiar with the organization’s culture and operational procedures. Collaboration and efficiency may both improve as a result. It might, however, limit team members’ skills and cause them to feel overworked or exhausted.

An independent game development team’s choice to use internal or external resources ultimately depends on its particular requirements and available resources. Combining the two may make sense, such as outsourcing some tasks while retaining the core team members on staff. This strategy can assist in striking a balance between the benefits of working with a team that is already familiar with the organization’s culture and procedures and the need for specialized knowledge and abilities.

Finally, having a team that supports and rallies around the initiative is critical. Putting together a team of high-profile individuals who don’t listen or are extremely eager to cause negative disruptions can jeopardize the overall effort. More time and effort should be invested in properly selecting and establishing the founding team, emphasizing the necessity of thorough hunting and attention to detail.

## 4. Orchestra

It is more difficult to keep a party together than it is to form one. Once a team is formed, the individuals involved must be given sufficient care and attention, as they are human beings with unique needs. The team’s success or failure is heavily reliant on effective people management. The capacity to effectively manage team members is critical in sustaining team cohesion and building a harmonious working environment, ensuring that everyone is aligned and working toward the same goal.

### 4.1 Relationships with co-founders/partners

**Figure 1.**
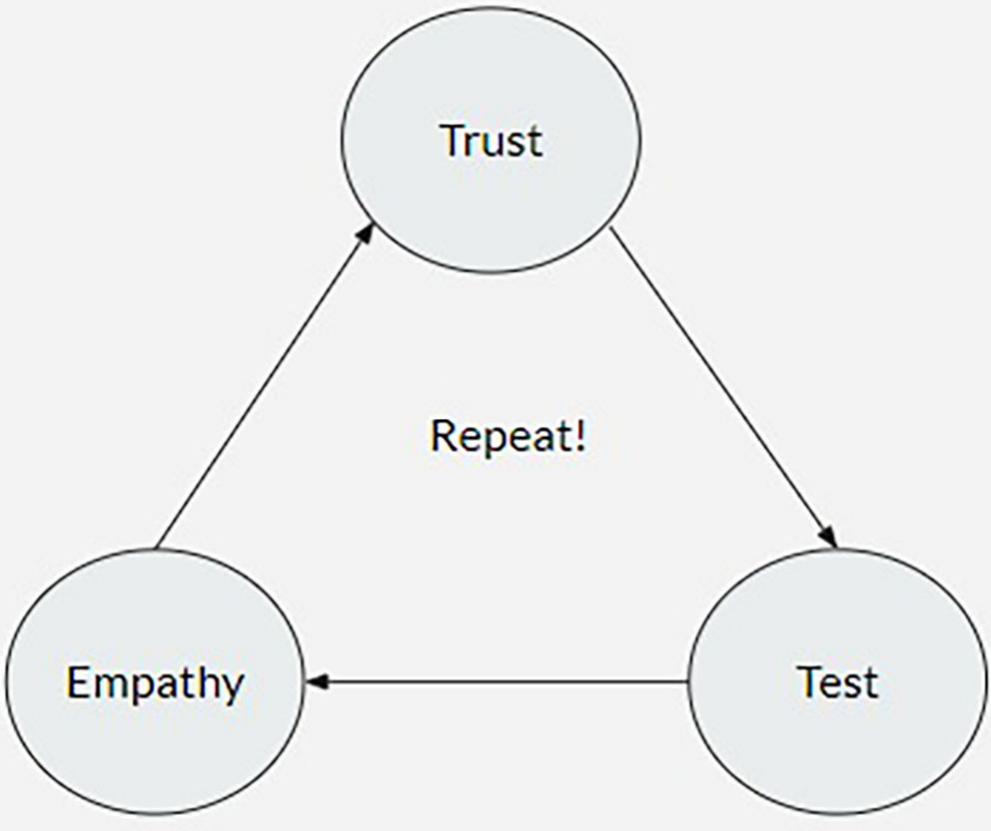
Chart showing the cycle of trust in a working partnership.

This relationship determines whether the team succeeds or fails. Depending on the circumstances, co-founders or partners can be a huge advantage or a significant detriment to the team and its mission. However, if a simple cycle is followed ( Figure 1), addressing this problem is not unduly complicated.

Any partnership starts with trust. People don’t form a business relationship unless they trust each other up to a certain level. It shouldn’t be disregarded or taken for granted, though. To ensure its performance, it needs regular inspections, much like routine maintenance. If managed improperly, problems could develop. As numerous academics have pointed out in the past, either online ( Weisband *et al*., 1995; Wellman *et al.*, 1996) or through physical interaction ( Brown & Duguid, 1991; Wenger, 1998), the motivating driver to the establishment of communities or networks can be found in common interests. These communities of interest represent a space where individuals’ interests and passions are orchestrated ( Wenger, 1998). Awareness and the desire for trust in independent teams go hand in hand. Al-Ani *et al*. (2013) claim that displaying socially acceptable behavior, technical proficiency, and consideration for others will increase trust among globally dispersed team members. Furthermore, it is noted that trust in remote creative teams is a dynamic process that changes during the work-life cycle. Wang and Redmiles (2016) found that informal, non-work-related contact (also known as cheap chat) among virtual team members in global teams (with the majority of members working remotely) was positively correlated with trust. There are several approaches to accomplishing this goal, but the most effective is to add regular timeout sessions for candid talks and open communication. It is frequently observed by us that these sessions operate best in informal settings rather than in a formal setting. Regardless, it is critical to have a consistent timetable for these discussions. Any potential difficulties or conflicts can be detected and handled during the maintenance phase. We have seen that partnership problems are frequently caused by a lack of empathy. Indeed, most problems may be traced back to a lack of empathy. As a result, encouraging and implementing empathy within the team is an effective strategy. Trust concerns are likely to be reduced greatly by developing an empathy culture. It can also be good to organize regular sessions dedicated to exercising empathy and understanding one other’s challenges. This observation is also supported by Frei and Morriss (2020), in their leadership case study, “ Begin with Trust”, the first step to becoming a genuinely empowering leader.

This iterative loop must be maintained throughout the collaboration. Sessions to assess empathy and trust may be held more frequently or less frequently depending on how long the company has been operating. Nevertheless, we feel that holding these workshops is a good idea. Given that prevention is so important, it is essential to recognize and solve any significant problems or fissures that may develop. By putting preventive measures into place, one can guarantee that should they ever be required, any cures will be simple. To maintain a strong, lasting, and harmonious relationship, communication between partners is crucial. Clarity must be prioritized, practiced, and integrated into the system in order to be effective. By doing this, a successful and long-lasting collaboration in business can be achieved.

### 4.2 Team chemistry

According to Villalobos (2011), developers place a great value on autonomy, and they are also accountable for managing their own work. For some developers, managing their time might become a significant problem in terms of completing their tasks. It is interesting to note how networking blogs and local events influence how developers approach their work. Although some creators like to retain a clearer distinction between the two, others favor using game jams to expedite their production.

Hence, team building, this task must be undertaken throughout the lifespan of the endeavor. It demands attention and care as long as there is a team. Individuals from various backgrounds, geographies, and ideologies are likely to make up the team. Even in paradise, difficulties are unavoidable. However, by taking a complete strategy to preserve smooth and dynamic team chemistry, not only will production increase, but internal difficulties will be reduced. Experts from many fields collaborate closely in a game development team, highlighting the need for rigorous work on team chemistry. The three Cs (compassion, commotion, and communication) are the keys to maintaining team cohesion.

The ability to comprehend and sympathize with the needs, emotions, and viewpoints of team members is a necessary component of compassion, as is the capacity to foster a welcoming and inclusive work environment. There are a few things that team members can do to show compassion for one another. First and foremost, a safe and supportive environment in which team members feel comfortable expressing their opinions and concerns in any situation should be fostered. This creates the groundwork for the formula’s second component: communication. We believe that it is also critical to embrace and celebrate diversity. According to a 2015 study of Harvard Business School alumni in senior management roles, 76% of respondents think that “a more diverse workforce improves the organization’s financial performance.” But academic studies have rarely discovered that greater diversity results in better financial performance. Under specific conditions, they have discovered that it results in higher-quality work, better decision-making, greater team happiness, and more equality ( Ely and Thomas, 2020). We noticed that each person brings their own set of talents, limitations, and work approaches to the table. Significant differences in mental processes are also prevalent. Significant progress may be made by recognizing and accepting these distinctions, as well as creating opportunities for team members to demonstrate their unique abilities and contributions. The team members will be motivated to advance if there are open lines of communication, open ears to their comments and recommendations, realistic goals, and recognition of their efforts and successes. If team members are overlooked, or do not have the essential support and resources to succeed, the repercussions can be terrible. Investing time and effort in building solid relationships with team members and providing them with enough resources, on the other hand, can result in enhanced productivity, creativity, and job satisfaction. The team’s well-being is critical to the success of an indie game development studio, underlining the need of sustaining and supporting them.

As a leader, it is critical to lead from the front. The leader should display compassionate behavior, establishing an example for others to follow. The leader does not have to be the most outspoken or disruptive member of the team. Rather, the leader can be someone who is well-liked by their team members, has clear views, and expresses them effectively. Travis Tracy ( How to Build a Game Development Team), who has led many indie teams, believes that a leader should be willing to take on unpleasant jobs, keep the team focused, show equal devotion to other team members, and be open to developing their technical skills. By being present, one must set a good example. Others will take the lead and be influenced. This idea applies to leadership in general as well as to this specific circumstance. It is necessary to be in charge when things matter. This should not, however, mean micromanagement. Instead, make an effort to be present when a team member marks a key religious or personal milestone. The entire team will be greatly impacted by such actions. Furthermore, it is crucial to set aside time to thank team members for their diligent efforts and vital contributions. This might be as simple as thanking them or highlighting their accomplishments at a team meeting. Members of the team should be gradually taught these concepts, so they may actively take part in the ritual of mutual celebration. Train them so they begin to follow this process as well.

We believe game development unit should be like a living organism, with team members that are comfortable with one another and eager to interact openly. While a rigid team structure may be appropriate for larger studios, the core of an indie team is found in the communal energy they generate. It is similar to a chemistry matchup in a game, where even if their characters aren’t the most powerful, they can aim to gain a competitive advantage by maintaining great chemistry among the team members. An indie team can achieve incredible feats in the area of game creation if they are motivated and interested. Do team members socialize outside of work? Is it common for them to spend time together playing games, watching movies, or dining out? Are they encouraging of each other’s personal achievements? Are their cohesiveness and coherence visible to spectators both close and far away? If these questions are answered affirmatively, it suggests that things are going well. It is critical to foster excitement, solidarity, and long-term partnerships. These connections will be invaluable when faced with tight deadlines, unanticipated challenges, and the danger of failure while still moving forward. In fact, these ties may be the deciding factor in overcoming adversity.

In a hypothetical team *vs* team encounter in a battle royale game, if a teammate fails to provide cover while a run for the bomb location is made, the opposing sniper will undoubtedly eliminate the player. The proper operation and eventual success of a team are dependent on excellent communication. However, delivering successful communication can be difficult and requires collaborative efforts from multiple viewpoints. Fostering a compassionate culture is a critical component in laying the groundwork for effective communication, functioning as a catalyst in achieving this goal. Team members should be eager to participate in open, thoughtful, and cooperative behavior with one another. Active listening should be practiced as well. A positive team culture supports equity because members have an open mind and show understanding and compassion for one another’s points of view. Active listening requires paying close attention to others, asking pertinent questions, and demonstrating empathy. Furthermore, communication ground rules should be developed and followed by team members. This step’s effectiveness will be determined by constant practice.

Because indie teams value freedom, the choice between working remotely and working from the office arises frequently. Working from home has been popular among many indie teams because of the ease it provides, allowing team members to work from the comfort of their own homes and providing greater flexibility in terms of work schedules. Working remotely has both pros and downsides. The greatest advantage of remote employment is the freedom it provides. Team members have the freedom to set their own schedules and move at their own pace, resulting in increased production and lower stress levels. Furthermore, working from home eliminates the need for a lengthy commute, which can save time and money. But there are some drawbacks to working from home as well. When working in the same environment, it may be difficult to keep work and personal life distinct. As a result, it may be challenging to concentrate and become distracted. Working remotely can often be lonely, which can hinder team members’ collaboration and communication.

In contrast, working at a workstation can give team members a focused location for work and aid in maintaining their concentration and productivity. The fact that everyone is in the same physical place might help team members collaborate and communicate more easily. Working at a workstation, however, cannot be as flexible as working from home and might include a commute, which can be expensive and time-consuming.

Additionally, not all team communications must take place in the same way. The ability of programmers to work together remotely has already been demonstrated. But evidence suggests that the creative design team may not be able to make it work. As an illustration, Bergström and Törlind (2007) examined the creative collaboration of design teams in both co-located and remote work contexts and found that the absence of shared collaborative capabilities (such as drawing surfaces) frequently led to the creative process being disturbed during distributed work.

Working from home or at a workstation will ultimately depend on the demands and preferences of the independent team. it is crucial to analyze the advantages and disadvantages of each choice and decide which would best support the team’s objectives, paying particular attention to communication between team members.

Every project’s success depends on the team’s ability to communicate effectively. To make sure that everyone is on the same page and is aware of what is expected of them, it is necessary to communicate messages clearly. Conciseness is essential for keeping messages concise yet complete and avoiding misconceptions or confusion caused by protracted explanations. Delivering constructive criticism, on the other hand, boosts productivity and teamwork while motivating team members to take responsibility for their work and pursue greatness. Communication can quickly deteriorate without the confidence to offer constructive criticism, resulting in mistakes and delays that could throw the entire project off course. Therefore, it is crucial to promote an environment of open and honest communication that values input and gives team members the freedom to give their all in pursuit of the group’s objective. It is crucial to maintain a quick tempo or to be cautious with a slower one in order to keep the group interested. It is best to wrap up the conversation as soon as possible while speaking with someone who is less knowledgeable. Hence we perceived that it is important to take into account all five senses that human possess in order to preserve a pleasant image with the team members.

Avoiding multitasking is crucial because it might impede effective communication. There is no place for the sixth sense in communication. The process of human communication is intricate and complicated, and depending solely on intuition or a “sixth sense” can have fatal results. In truth, this method of communication is similar to gambling in that it might succeed once, but the chances of failure are very high. it is important to continue with tried-and-true communication techniques that are based on objectivity and clarity. It is possible to make sure that our message is delivered accurately and effectively by avoiding ambiguous wording or assumptions. In other words, rather than relying on intuition or guesswork, effective communication relies on precise facts and understandable language. Inadequate communication can have disastrous repercussions, including crying and a host of other negative results.

A skilled communicator should be developed or hired to support the team’s successful emergence because communication acts as the glue holding the group together.

### 4.3 Conflict resolution

Team members may still become involved in sporadic confrontations even after taking the aforementioned safeguards, as they happen more frequently than is normally anticipated. It is advantageous to therefore plan ahead for post-conflict measures as well.

Recognizing and acknowledging the presence of a problem is the first step in its settlement. Denial is a common reaction to conflict, especially in the early phases. While denial may be a strategic choice for handling a specific dispute at a certain time, in most cases, it is an unsuccessful technique that ignores or dismisses the appearance of a possible conflict. Most of these difficulties can be resolved quickly if they are given adequate attention. By default, there is a propensity to trust that the problem will resolve itself, which ultimately worsens matters in the long run. Acknowledgment of the concerns must first come from the parties involved, and then it may be open to acknowledgment by other relevant parties, if applicable.

There are various strategies for settling a problem amicably. It will depend on how the conflict is going and what stage it is at. According to Alistair Doulin (2010), the best choice for resolving conflicts is to have someone in a position of authority. This person must be believed not to advance their personal interests. The best and simplest way to handle problems is through open, continuous communication between team members, who can speak things out. It becomes harder to please everyone as the team size increases. A compromise is necessary. The team is in it together, thus disagreements must be resolved through compromise. Voting is a great way to resolve conflicts since it essentially offers everyone a voice. The team must accept and respect the use of a voting method. Many confrontations can be efficiently resolved before they escalate if all parties involved acknowledge and communicate openly. Taking a mutually agreed-upon break might also be beneficial in some instances. If the office has a competent HR department, it may be appropriate to assign the conflict resolution process to them. In this setting, the function of human resources is critical. If they have earned the trust of their staff and have built positive relationships with everyone, they will be aware of such concerns. One could wonder if accepting this responsibility personally is a good idea. There is no harm in accepting the post if one holds the essential competence and is judged the best appropriate for it. However, team members may be cautious to fully reveal their opinions and concerns if they believe the individual is the ultimate decision-maker. Individuals frequently overthink and hide facts for fear of being viewed as unimportant. However, if one builds a position of trust and true camaraderie with the team, they may be able to effectively handle the function on their own. It is worth noting that many game developers are introverted people who have a deep passion for their art. Without developing a close bond with them, they may be selective in sharing personal information or secrets. The conditions for developing trusting links and reinforcing shared beliefs aims, and practices are created *via* reciprocal relationships and shared experiences as a community of practice and production ( Pargman, 2005: 106). Independent creators recognize their strengths through community interaction and sharing performance synergies. Most disputes go unreported by the people concerned. They can let issues develop and worsen until they become unbearable. As a result, it is critical to provide sufficient care for the team while remaining watchful and cultivating a pleasant relationship between the HR department and the team members. This strategy will help to improve conflict resolution and overall performance.

### 4.4 Long-term assets: look after your nukes

We will start this section with an example first. Nuclear warheads with the ability to inflict significant damage across a large region are available in the Command and Conquer series, a popular choice among fans of real-time strategy games. The weapons such as nukes have the inherent ability to have a big impact on the game’s final outcome. However, an important barrier emerges: nuke’s creation takes a significant amount of time, which is exacerbated by the lengthy period required for them to become operational. Throughout this extended period, the warheads remain highly vulnerable to attack. If someone’s adversary has a stealth unit, their tactical approach may include breaching their defenses and grabbing the warhead before the nukes achieve full readiness, then using it against their soldiers. The occurrence of such a situation as a competitor coveting a well-trained resource for game development in one’s professional life can have enormous ramifications, especially when dealing with raw abilities that are sought for, nourished, and cultivated. These people frequently have enormous potential that can be achieved over time. When a firm nurtures a strong culture and delivers high-quality internal education, the industry as a whole takes note and prepares itself as these individuals reach their full potential. Since organizational culture defines the environment for everything that happens within a company, one should try to focus and maintain this aspect ( What is organizational culture and why is it important). However, if these talents do not have strong ties to the business, they risk being stolen away just as they are about to pay back the investment placed in them. While attending Gamescom Asia 2022 which was held in Singapore, multiple game industry insiders while conducting sessions shared that game development companies in Singapore facing recruitment difficulties due to severe rivalry. In an atmosphere where large corporations coexist, presenting appealing services and aspirations, small studios face a tremendous challenge. However, it is critical to capitalize on one’s abilities and focus on things that large corporations frequently struggle to supply. Being a part of an indie team implies a unique environment marked by independence, enthusiasm, a high learning curve, and recognition. It is critical to ensure that young talents understand the risks they are taking by joining the team and can see their goals being realized while remaining with the club as they advance. Constant monitoring and unflinching care are required, never allowing the young talents to escape sight and protecting them with all available means. Otherwise, a potentially groundbreaking decision could devolve into a nightmare. While it is conceivable to substitute a ready-made resource to some extent, it is impossible to replace someone personally trained with someone who was trained by someone else. They will never be identical, which will have irreversible effects if they are lost.

### 4.5 Master the Roguelites: retain the process

Roguelite games are difficult to master since success is rarely obtained on the first try. Playing, failing, and trying again become necessary steps in this process. Each failure teaches and rewards the learner, enhancing one’s talents for future attempts. This iterative process must be repeated until victory is achieved. It is quite common for assembling an amazing team to necessitate extensive fine-tuning and constant effort, in a dynamic that could be seen to mirror these games. It is critical not to be hesitant in parting ways with individuals who are destructive to the culture of your team. In the long run, their existence may hinder the voyage of other valuable assets. While people come and go, the process is unchanging. It is critical to develop and adopt a systematic approach to all aspects. Proper boundaries and clarity should be established within the framework of game production, a multidisciplinary art form that requires the collaboration of people from many backgrounds and viewpoints. A programmer may be oblivious to the time and work necessary by a concept artist to develop a simple character in five hours, just as others may wonder why a coder spends days polishing a basic leaping function. This is why cultivating an empathy culture is critical since it allows people to understand and accept one another’s difficulties and processes. It is also critical to handle creative resources with care and sensitivity. They should not be overworked, but some work-related ground norms and practices must be established and enforced. Their creative freedom, however, should never be compromised. Everyone in an indie team with a strong culture can find excitement in every area of game production and have an opinion on a variety of issues. Nonetheless, specific choices should be delegated to specific personnel. The narrative lead has ultimate power over the story’s conclusion, whereas the lead game designer assesses whether the win condition is biased. Team members are encouraged to share their opinions and provide constructive input, but harmful language should be avoided. Individuals with substantial gaming experience, having played over 1000 games in their lifetime, may be present on the squad. While their feedback is crucial, it is critical to ensure that their decision-making authority remains within and does not exceed the stated bounds. Furthermore, they should not engage in bullying conduct in which they harass others based on their personal preferences, referring to games that they have only played. Setting these boundaries will assist the team in avoiding a variety of undesired scenarios and fostering a successful collaborative environment.

## 5. Conclusion

Creating an independent team can be difficult yet rewarding for game developers. Developers can choose the most effective strategy for their project by weighing the benefits and drawbacks of various team arrangements. The techniques previously described are all the outcomes of navigating the process of creating one’s own independent teams. Independent teams can overcome the challenges brought on by scarce resources with proper preparation and clear communication.

In conclusion, it is crucial to begin with a clear knowledge of the project’s vision, goals, and scope in order to assure a successful independent team creation. Developers can then select whether to outsource or use internal personnel after identifying the roles and talents that their team needs.

Once the team is established, productive collaboration depends on effective communication. The team can stay on track by setting clear goals, scheduling frequent check-ins, and using tools like project management software and video conferencing.

Putting together an independent team involves careful thought and preparation, but with the correct attitude, it can result in the creation of creative and commercially successful games. It is unquestionably important to think about the various questions that are raised and decide what best piques your attention.

When embarking on the adventure of establishing an indie team, it is critical to remember that team formation is similar to the establishment of a personal Justice League, albeit without superpowers. The hunt is looking for someone with sufficient skills in programming, design, art, and communication. Nonetheless, it is critical to remember that exceptional team creation entails the heavy burden of sustaining and growing that team. The journey ahead will likely provide its fair share of challenges and hurdles, as with every major trip. However, with a well-defined vision, a solid plan, and a team of experienced and devoted individuals, there are no impediments to realizing dreams of creating the next innovative indie game sensation. Thus, go forth and conquer the world of indie game creation!

## Data Availability

No data are associated with this article.
