# Supplementary material for: Building Your Dream Team: How Indie Teams Can Form, and Thrive Together
Source: F1000Res. 2024 Jan 8;13:42. [Version 1] doi: 10.12688/f1000research.139274.1 (PMC11069039; doi:10.12688/f1000research.139274.1)
Supplement: Peer review history

# Reviewer report: Reviewer response for version 1

**DOI:** 10.5256/f1000research.152539.r266634

**Published:** 2024-05-03

**Authors:** Matt Barton

**Affiliations:** 1 St Cloud State University, Saint Cloud, Minnesota, USA

I enjoyed this article and found it well-researched and clearly written. I think it will be helpful for anyone considering forming or joining an indie team and ensuring a smoother collaboration. As far as weaknesses go, I noticed one spot where "my experience" is used, which is confusing considering there are two authors on this piece. Another spot needing attention is the "three c's," which mentions "commotion." I'm not sure if that is the right word there; if it is, it would be helpful if the authors defined what that means in this context. Finally, although there are a few good real-life examples included (such as the Singapore situation), it'd be good to have more anecdotes throughout the piece to exemplify the value of the points being discussed. Perhaps a fictional indie team could be introduced to illustrate the principles, such as the potential impediment of multitasking during team sessions? Overall, though, I think the article is thoughtful and certainly addresses some of the key issues faced by indie studios.

Is the topic of the opinion article discussed accurately in the context of the current literature?

Yes

Are arguments sufficiently supported by evidence from the published literature?

Yes

Are all factual statements correct and adequately supported by citations?

Yes

Are the conclusions drawn balanced and justified on the basis of the presented arguments?

Yes

Reviewer Expertise:

Professional communication, game studies

I confirm that I have read this submission and believe that I have an appropriate level of expertise to confirm that it is of an acceptable scientific standard.

# Reviewer report: Reviewer response for version 1

**DOI:** 10.5256/f1000research.152539.r238205

**Published:** 2024-02-15

**Authors:** Josiah Lebowitz

**Affiliations:** 1 George Mason University, Fairfax, Virginia, USA

**Summary**

 The article discusses the challenges of creating and maintaining a team for indie game development. It divides the process into several steps, with the main focuses being first on finding and hiring appropriate team members, and then ensuring a productive and harmonious working environment for the newly formed team.

**Is the topic of the opinion article discussed accurately in the context of the current literature?**

 Yes, the material is accurate and suitably reflects current trends, literature, and the like.

**Are all factual statements correct and adequately supported by citations?**

 Yes, the article is well cited.

**Are arguments sufficiently supported by evidence from the published literature?**

 Yes, sufficient evidence is provided when needed.

**Are the conclusions drawn balanced and justified on the basis of the presented arguments?**

 Yes, the conclusions are reasonable and build well on the presented material and arguments.

**Comments/Suggestions**

 - Section 2.4 Paragraph 2: Inconsistent formatting is used when listing the different team roles. 

 - Section 2.4 Paragraph 2: Both the writer and designer are listed as being in charge of the story. This can be confusing without some clarification. Depending on the team makeup, it might be entirely under the writer’s control. Alternately, the lead designer or creative director could create the general story summary with the writer being in charge of the full script. There are also many situations, especially in indie development, where the designer and writer will be merged into a single role (narrative designer) with the responsibilities of both positions.

 - Section 2.4 Paragraph 2: It’s worth mentioning that, depending on the game engine and type of game, a programmer might not be necessary. While relatively uncommon, there have been some notable indie games that were created entirely without a programmer using only the engine’s built-in tools.

 - Section 2.4 Paragraph 3: The brief mention of a spouse should be removed. While having a spouse or significant other of some type is invaluable for many people’s emotional and physical wellbeing (indie developers and otherwise), it doesn’t really fit the theme of the paper and isn’t the type of thing that should be discussed in a paper like this without significant detail and supporting documentation.

 - Section 3.2 Paragraph 1: While it may vary somewhat in different parts of the world, North America is starting to see a large number of recent graduates with rigid attitudes and strongly held social and cultural beliefs which can lead to (sometimes significant) problems when they are hired. As such, it’s worthwhile to look for appropriate warning signs in all applicants, not just industry veterans.

Is the topic of the opinion article discussed accurately in the context of the current literature?

Yes

Are arguments sufficiently supported by evidence from the published literature?

Yes

Are all factual statements correct and adequately supported by citations?

Yes

Are the conclusions drawn balanced and justified on the basis of the presented arguments?

Yes

Reviewer Expertise:

Game Design, Narrative Design, Creative Writing & Storytelling, Interactive Storytelling, Game Development, Indie Game Development

I confirm that I have read this submission and believe that I have an appropriate level of expertise to confirm that it is of an acceptable scientific standard.
